# Effectiveness of the First German-Language Group Psychotherapy Manual to Accompany Short-Term Treatment in Methamphetamine Dependence

**DOI:** 10.3389/fpsyt.2020.00130

**Published:** 2020-02-28

**Authors:** Johannes Petzold, Benjamin Weber, Tyler Ray Bassett, Michael Bauer, Nadine Bernhardt, Cornelius Groß, Helena Hasler, Matthias Schützwohl, Maximilian Pilhatsch

**Affiliations:** Department of Psychiatry and Psychotherapy, Carl Gustav Carus University Hospital, Technische Universität Dresden, Dresden, Germany

**Keywords:** methamphetamine, psychotherapy, manuals, effectiveness, prognosis, predictors, Germany

## Abstract

**Background:** Methamphetamine abuse is expanding in Europe, leading to a shortfall in medical care for related disorders in many regions. Research focusing on the effectiveness and feasibility of methamphetamine-specific treatment programs is scarce, especially in short-term settings.

**Methods:** To this end, we treated 31 patients with methamphetamine dependence using a new group psychotherapy manual added to standard psychiatric care. Trained research assistants recorded demographic, illness and treatment variables using a standardized interview at baseline and a follow-up visit 3 months later. Outcome and process variables for this intervention encompassing 15 modules for qualified detoxification and motivation of patients with methamphetamine dependence are reported.

**Results:** Retention and abstinence rates as well as acceptance and feasibility in daily routine were assessed positively. Patients with an unsuccessful outcome were characterized by longer regular methamphetamine use (*t* = −2.513, df = 29, *p* = 0.018) and a shorter abstinence period at baseline (U = 74.500, *z* = −1.808, *p* = 0.072). Among the demographic and clinical variables, the only predictor significantly increasing the odds of a successful outcome was a shorter period of regular methamphetamine use (OR = 1.318, CI 95% for OR = 1.021–1.700, b = 0.276, SE = 0.130, *p* = 0.034).

**Conclusions:** This freely available therapy manual can help counter the shortfall in available psychotherapeutic interventions for patients with methamphetamine dependence in German-speaking countries. The routinely assessed parameters duration of regular methamphetamine use and abstinence before treatment were associated with outcome and may be used to personalize therapeutic strategies.

## Introduction

Methamphetamine dependence is a growing global problem with major regional differences. Due to these differences, the availability of methamphetamine has affected certain cultures more than others. The annual prevalence of (meth-)amphetamine use in the European general public was 0.7% in 2012, with adults between the ages of 25 to 29 exhibiting a 12-month prevalence as high as 2.4% (1). Care providers in the German states of Bavaria, Saxony, Saxony-Anhalt and Thuringia (2) were particularly challenged in the past decade (3) due to the proximity of these states to the Czech Republic, which has a comparatively long history of methamphetamine consumption and production (5–10 tons per year) (4). A meta-analysis has shown that psychosocial interventions for substance use disorders are effective (5), yet the transferability of these interventions for the treatment of methamphetamine dependence may be limited due to rewarding effects concerning sexual drive (6), euphoria (7) and a greater likelihood for developing major psychiatric disorders compared with other drugs (8). However, there has been little research focusing on the effectiveness and feasibility of methamphetamine-specific treatment programs.

In the German healthcare system, treatment of drug-related disorders is divided into acute therapy, i.e., inpatient qualified withdrawal treatment for 3–4 weeks, and seamless post-acute management spanning from 3 to 12 months delivered either as outpatient, semi-inpatient or inpatient treatment (8). In post-acute long-term settings, the Matrix Model—a methamphetamine-specific, group-based treatment program consisting of cognitive behavioral therapy, family therapy and self-help sessions—demonstrated higher retention and completion rates than non-specific inpatient post-acute management programs (9). Following this rationale we adopted and translated the manual by Lee et al. (10), which is in line with the quality standards recommended by the German treatment guidelines for methamphetamine-related disorders (8). The original manual has been proven effective in treating methamphetamine use disorders compared with other treatment options (11), and the version adapted by our department has demonstrated good feasibility in daily clinical routine (12).

The aim of the present study was to evaluate the effectiveness of the first German-language group psychotherapy manual specifically developed for treating patients with methamphetamine dependence, using a short-term approach in a real-world setting. Based on other studies focusing on the Matrix Model, we expected similar retention rates of approximately 40%. Moreover, we explored if participant characteristics at baseline were associated with outcome to identify predictors for a successful treatment.

## Methods

This is a real-world study testing the effectiveness of the first German-language group psychotherapy manual using short-term treatment in patients with methamphetamine dependence. The methods were performed according to relevant national and institutional guidelines and regulations as approved by the institutional review board of the Technische Universität Dresden. All participants gave written informed consent in accordance with the Declaration of Helsinki.

### Intervention

The German manual is available free of charge from the corresponding author and has been described in detail elsewhere (12). In summary, it features a methamphetamine-specific relapse prevention program based on motivational interviewing, which comprises 15 modules: [1] introduction to treatment rationale including functional analysis of last methamphetamine consumption, [2+6] motivational clarification and enhancement (if necessary also in further course of therapy), [3−5] understanding and managing of cravings, [7−9] the role of the social environment and role-play training of strategies to resist drug use, [10+11] awareness of seemingly irrelevant decisions, [12+13] identifying and dealing with other high-risk situations, [14] drafting a personal crisis plan, [15] coping with problems after the end of therapy. The 15 sessions lasted 50 min each and were delivered to groups of up to 6 participants by a psychotherapist twice a week. Patients could join the program at any module, but received an individual session before in which the program was introduced.

### Study Procedure

To ensure a naturalistic study sample, all patients using either an in- or outpatient treatment between the ages of 18 to 65 diagnosed with methamphetamine abuse or dependence according to the International Classification of Diseases (ICD-10) were offered study participation. Patients were eligible to participate after symptoms of intoxication and withdrawal subsided and they maintained abstinence from any drug for at least 2 days, proven by negative urine results (Drug-Screen Multi 4TL-AC-A, nal von minden, Moers, Germany) for amphetamines, methamphetamines, methylenedioxymethamphetamine (MDMA), opioids, tetrahydrocannabinol (THC), benzodiazepines and tricyclic antidepressants. Any condition (e.g., psychotic or severe affective symptoms, cognitive impairment and reduced mobility) that would have interfered with the capability to attend group psychotherapy led to exclusion from the study.

Trained research assistants recorded demographic, illness and treatment variables using a standardized interview at baseline and a follow-up visit approximately 3 months later. Outcome was classified as successful if patients (a) attended at least 8 out of 15 group sessions or enrolled in a post-acute management program and (b) had no more than 1 methamphetamine relapse until the follow-up visit indicated by drug tests, provided the relapse was admitted and self-critically processed. Inpatients were tested unannounced at least once a week, whereas outpatients were randomized unannounced with a 1/6 probability of urine screens between Monday and Friday. Urine was collected under direct observation followed by temperature measurement to minimize manipulation.

### Statistics

We used SPSS Statistics 25 (IBM, Armonk, NY, USA) and assumed 2-tailed significance at *p* < 0.05 for all analyses. All tests were based on the whole study sample (*N* = 31) and data were complete on all implicated variables (i.e., no missing values). Histograms and normal quantile-quantile plots were used to judge normality. Descriptive analyses were conducted to characterize demographic, illness and treatment variables as well as treatment outcome. We compared participant characteristics and the number of group sessions attended according to treatment outcome (successful vs. unsuccessful) using Pearson's chi-square test for categorical variables, applying Fisher's exact test if needed and the independent *t*-test for continuous variables, applying the Mann Whitney *U*-test if needed. Variables that considerably differed between outcome groups (successful vs. unsuccessful) were correlated across groups and reported as Pearson's r or Spearman's ρ as appropriate. To identify predictors for a successful outcome, all participant characteristics reported in Table 1 were explored by logistic regression analyses with forward stepwise selection, using natural log transformations of abstinence durations at baseline to meet the assumptions of parametric testing.

**Table 1:** Participant characteristics at baseline.

	**Successful outcome**	**Unsuccessful outcome**	**Statistics**
Sample size	15 (48.4)	16 (51.6)	
**Demographics**			
Sex			X^2^ = 4.045, df = 1, *p* = 0.044*
Women	8 (53.3)	3 (18.8)	
Men	7 (46.7)	13 (81.3)	
Age [years]	27.53 ± 5.29	30.75 ± 7.73	*t* = −1.343, df = 29, *p* = 0.190
Romantic relationship	7 (46.7)	6 (37.5)	X^2^ = 0.267, df = 1, *p* = 0.605
Children	9 (60.0)	10 (62.5)	X^2^ = 0.020, df = 1, *p* = 0.886
Lower secondary school leaving certificate or less	9 (60.0)	14 (87.5)	X^2^ = 3.058, df = 1, *p* = 0.113
Unemployed	9 (60.0)	13 (81.3)	X^2^ = 1.697, df = 1, *p* = 0.252
**Clinical data**			
Methamphetamine dependence	15 (100.0)	16 (100.0)	
Age of first methamphetamine use [years]	18.20 ± 4.31	19.81 ± 5.91	*U* = 139.000, z = 0.760, *p* = 0.470
Regular methamphetamine use [years]	5.17 ± 2.58	8.69 ± 4.81	*t* = −2.513, df = 29, *p* = 0.018*
Abstinence [days]	33.73 ± 69.23	5.63 ± 4.50	*U* = 74.500, *z* = −1.808, *p* = 0.072
Abstinence confidence for the next 3 months^A^	8.07 ± 2.09	7.06 ± 1.73	*U* = 82.000, *z* = −1.534, *p* = 0.140
Current psychiatric comorbidity^B^	10 (66.7)	7 (43.8)	X^2^ = 1.642, df = 1, *p* = 0.200
First or second degree family history of mental disorders^C^	7 (46.7)	6 (37.5)	X^2^ = 0.267, df = 1, *p* = 0.605

All tests are based on the whole study sample (N = 31) and complete data on all variables. Data are number (%) or mean ± SD.

A Likert scale, from 1 = not very safe to 10 = very safe,

B according to ICD-10,

C according to (15), *significant at p < 0.05.

## Results

### Participant Flow and Retention

We offered study participation to 48 in- and outpatients from the Carl Gustav Carus University Hospital in Dresden, Germany of which 10 patients were not interested and 7 patients did not meet the eligibility criteria. Of the 31 patients enrolled from March to December 2017, 15 were classified as having a successful outcome (48.4%), 5 of whom were referred to a post-acute management program before completing group psychotherapy. Twenty of 31 participants attended the follow-up visit (64.5%), including 3 of the referred patients and 7 of the 16 participants who were classified as having an unsuccessful outcome. Patients with successful outcome participated in more group psychotherapy sessions (mean ± SD = 8.33 ± 5.21, without referred patients: 10.10 ± 5.32) than patients with an unsuccessful outcome (4.88 ± 2.99) (U = 69.000, *z* = −2.028, *p* = 0.045, test based on *N* = 31).

### Participant Characteristics and Outcome Prediction

Demographic and clinical characteristics were comparable between patients with a successful and with an unsuccessful outcome, except for sex distribution and the duration of regular methamphetamine use (see Table 1). Current psychiatric comorbidities were depressive episodes (*N* = 6), harmful use of THC (*N* = 5), alcohol dependence (*N* = 4), attention-deficit hyperactivity disorder (*N* = 4), drug-induced psychotic disorder (*N* = 3, subsided before start of psychotherapy), borderline personality disorder (*N* = 2), dissocial personality disorder (*N* = 1), posttraumatic stress disorder (*N* = 1) and schizophrenia (*N* = 1). While sex was almost equally distributed in the group with successful outcome, participants with an unsuccessful outcome were predominantly male (81.3%) and characterized by longer regular methamphetamine use (*t* = −2.513, df = 29, *p* = 0.018) and a shorter abstinence period at baseline (U = 74.500, *z* = −1.808, *p* = 0.072). Correlational analysis revealed that regular methamphetamine use was longer in men across groups (*r* = 0.362, *p* = 0.046). Although only reaching trend-level significance, we were interested in whether such a relationship also existed for abstinence. However, abstinence period was not significantly correlated with sex (ρ = −0.083, *p* = 0.656; numerically shorter in men) or with the duration of regular methamphetamine use (ρ = −0.116, *p* = 0.535) across groups. Among the demographic and clinical variables, the only predictor significantly increasing the odds of a successful outcome was a shorter period of regular methamphetamine use (OR = 1.318, CI 95% for OR = 1.021–1.700, b = 0.276, SE = 0.130, *p* = 0.034).

## Discussion

We aimed to evaluate the effectiveness of the first German-language group psychotherapy manual specifically designed for short-term treatment in patients with methamphetamine dependence in a real-world setting. Almost half of the participants were classified as having a successful outcome, which was based on abstinence, group psychotherapy attendance and enrollment in a post-acute management program. Women were found to be significantly more successful compared to men with shorter periods of regular methamphetamine use at baseline than those with an unfavorable outcome. A shorter period of regular methamphetamine use was the only significant predictor of a successful outcome.

### Methamphetamine-Specific Psychotherapy

A comparison between short- and long-term interventions led to heterogeneous results. Additionally, differentiation between in- and outpatient settings is of interest. Concerning interventions for methamphetamine-related disorders, observation periods varied between 3 weeks and 6 months, with retention rates between 90% (9, 13, 14, 16) and 30% (17). In China, Srisurapanont et al. (16) enrolled 48 students with methamphetamine abuse or dependence in a randomized controlled trial in an outpatient setting comparing a methamphetamine-specific brief intervention consisting of two 20-min sessions with one 15-min psychoeducation session. The brief intervention appeared to reduce the days of methamphetamine use at the 8-week endpoint (1.97 days to 1.09 days, *p* = 0.04). Dropout rates were 50 and 30% for the brief intervention and psychoeducation group, respectively. The most studied methamphetamine-specific outpatient program is the cognitive behavioral therapy based 16-week Matrix Model (18). Whereas retention rates did not statistically differ between the Matrix Model (65%) and a complex inpatient treatment routine based on a therapeutic community model (51%) in 115 patients in Thailand (19), a higher short-term retention was achieved in the US comparing the Matrix manual with non-methamphetamine-specific treatment as usual in 978 patients (9). Of note, this superiority leveled off in 2 post-treatment time points. Recently, a German work group found no significant differences in dropout rates (41% across groups) between 2 inpatient programs for post-acute management of 108 patients with methamphetamine abuse (20). Interestingly, retention rate was higher (*p* = 0.001) in the treatment-as-usual group (171 vs. 128 days), but this may be due to structural differences between treatment facilities assuming a better treatment allegiance in the treatment-as-usual facility (20). On a broader scale, a Cochrane review including 52 trials (6,923 participants) reported a dropout rate of 32% for all individuals involved in psychostimulant misuse (21). Nevertheless, because of the heterogeneity of the results, it is unlikely that there is a one-size-fits-all approach. With this in mind, the present study supports the finding in contemporary substance abuse literature that all treatment conditions are associated with comparable levels of improvement.

### Predictors of Favorable Outcome

A systematic review including 199,331 participants of 122 addiction treatment studies concluded that consistent predictors for dropout were younger age, personality disorders and low treatment alliance, with younger age being the most robust predictor (22). It is important to emphasize that research focusing on stimulant use disorders has been scarce. In cocaine dependence (*N* = 286), younger patients who did not complete high school and had more days of cocaine use in the previous month were less likely to complete 1 week of abstinence at the beginning of treatment (23). In another study, drug use variables did not predict time to dropout, but younger African American patients and unemployed patients were more likely to drop out earlier of psychosocial treatment for cocaine dependence (24). Furthermore, psychiatric severity was associated with women dropping out sooner but not in men. In methamphetamine dependence, injection drug use (20), history of drug injecting, employment status and multiple sexual partners (25) were identified as predictors for an unfavorable outcome. We did not assess drug injection use or sexual behavior in our study, but age, being in a romantic relationship or employed did not considerably differ between outcome groups. By contrast, methamphetamine use before treatment was longer in patients with an unsuccessful outcome and was also found to be a significant predictor. Of note, those patients were predominantly male with substantially longer regular methamphetamine use in men across groups. Since abstinence period at baseline tended to be shorter in these patients without being significantly correlated with sex across groups, sex and drug use variables seem to have at least a partially independent influence on treatment success. Taken together, our data indicate that a longer duration of regular methamphetamine use and a shorter abstinence period before treatment are linked to a more difficult course of treatment. As these parameters are easy and take little time to collect, they can be utilized to identify specific patients, who may benefit from this manual or are even at risk for an unsuccessful outcome. If necessary, individual treatments could then be escalated quickly, e.g., through augmenting the program with individual psychotherapy sessions.

The different predictors found across studies may be explained by differences in sample characteristics, possibly reflecting country-dependent patterns of addiction or broader cultural themes. Of note, our study may have lacked statistical power to detect predictors of smaller effect sizes. Moreover, we did not test for differences in single psychiatric comorbidities between outcome groups due to an insufficient number of cases.

### Strengths and Limitations

To our knowledge, this work represents the first study evaluating the effectiveness of a German-language group psychotherapy manual to accompany short-term treatment in patients with methamphetamine dependence. The study of a naturalistic sample demonstrated the transferability of this program into the real world. Given the external validity, our findings concerning clinical markers are of direct importance for day-to-day patient care. Yet since country-dependent epidemiological research addressing consumers and their consumption patterns is non-existent, our results may not be generalizable. Moreover, our study is limited by a rather small sample (*N* = 31), the lack of a control group with a non-methamphetamine-specific intervention and a follow-up restricted to 3 months.

## Conclusion

We positively evaluated the effectiveness of the first German-language group psychotherapy manual to accompany short-term treatment in patients with methamphetamine dependence in a real-world setting. The manual is available free of charge from the corresponding author and can be implemented easily into the daily clinical routine and help counter the shortfall in available psychotherapeutic interventions for patients with methamphetamine dependence in German-speaking countries. Male patients and patients with a longer regular methamphetamine use before treatment should receive particular attention when applying this program as these factors were associated with an unfavorable outcome. Since treatment success was only 48% similar to other psychological interventions with different outcome predictors associated with different programs, it emphasizes that we still know little about what works for whom. Large-scale head-to-head studies are needed to further our understanding of the active ingredients of psychological interventions for patients with methamphetamine dependence.

## Data Availability Statement

The datasets generated for this study are available on request to the corresponding author.

## Ethics Statement

The studies involving human participants were reviewed and approved by the institutional review board of the Technische Universität Dresden. The patients/participants provided their written informed consent to participate in this study.

## Author Contributions

MP and MS designed the study. MP obtained funding. CG delivered group psychotherapy. CG, HH, BW, and MP collected the data. JP analyzed the data, wrote the methods and results and contributed to the introduction and discussion. JP, MP, BW, NB, MB, and TB interpreted the data. BW wrote the introduction and discussion. MP, TB, NB, MS, MB, CG, and HH revised the manuscript critically for important intellectual content. All authors read and approved the final manuscript.

### Conflict of Interest

The authors declare that the research was conducted in the absence of any commercial or financial relationships that could be construed as a potential conflict of interest.
